# UV-Based Advanced Oxidation Processes of Remazol Brilliant Blue R Dye Catalyzed by Carbon Dots

**DOI:** 10.3390/nano12122116

**Published:** 2022-06-20

**Authors:** Inês M. F. Cardoso, Rita M. F. Cardoso, Luís Pinto da Silva, Joaquim C. G. Esteves da Silva

**Affiliations:** Chemistry Research Unit (CIQUP), Institute of Molecular Sciences (IMS)–DGAOT, Faculty of Sciences of University of Porto (FCUP), Rua do Campo Alegre s/n, 4169-007 Porto, Portugal; up201704720@edu.fc.up.pt (I.M.F.C.); up201704723@edu.fc.up.pt (R.M.F.C.); luis.silva@fc.up.pt (L.P.d.S.)

**Keywords:** textile dyes, Remazol Brilliant Blue R, reactive blue 19, UV based AOP, nanomaterials, carbon dots

## Abstract

UV-based advanced oxidation processes (AOPs) (UV/H_2_O_2_ and UV/S_2_O_8_^2−^) with a titanium(IV)-doped carbon dot, TiP-CD, as a catalyst were developed for the decomposition of Remazol Brilliant Blue R (Reactive Blue 19), an anthraquinone textile dye (at T = 25 °C and pH = 7). The Ti-CD, with marked catalytic UV properties, was successfully synthesized by the one-pot hydrothermal procedure, using L-cysteine as carbon precursor, ethylenediamine as nitrogen source, PEG (polyethylene glycol) as a capping agent, and titanium(IV) isopropoxide (precursor of TiO_2_ doping). Contrary to azo dyes (methyl orange, orange II sodium salt, and reactive black 5), which achieved complete degradation in a time interval less than 30 min in the developed AOP systems (UV/H_2_O_2_, UV/S_2_O_8_^2−^, and UV/TiO_2_), the RBB-R showed relatively low degradation rates and low discoloration rate constants. In the presence of the catalyzer, the reaction rate significantly increased, and the pseudo-first-order rate constants for the RBB-R discoloration were UV/3.0 mM H_2_O_2_/TIP-CD-0.0330 min^−1^ and UV/1.02 mM S_2_O_8_^2−^/TIP-CD-0.0345 min^−1^.

## 1. Introduction

The textile industry is one of the most polluting industries in the world, consuming 93 billion cubic meters of water. In fact, 20% of wastewater worldwide comes from fabric dyeing and treatment [1,2]. The increase of fast-fashion trends complicates the environmental impact of textile business, and the lack of garment eco-design inhibits efficient circular economy strategies [3]. About 80% of the global emissions of this industry are discharged into environmental water, and, although textile effluents are constituted by a huge number of chemical substances, natural fibers, and microplastics, of greater concern are the large amount of non-biodegradable organic compounds, especially textile dyes [4,5]. The treatment of these substances in wastewaters requires advanced oxidation processes (AOPs) [6]. One of the types of AOPs that can be used for the degradation of selected pollutants present in wastewater is based on ultraviolet light (UV); that is a standard technology for water disinfection, but it can also be coupled to several other chemical systems, such as, for example, hydrogen peroxide and persulfate anion, enhancing the performance in AOP [7,8].

The direct exposition of a dye (Dy) to high-energy UV light creates an excited state molecule (^#^Dy) or the photolysis of a chemical bond, resulting in its fragmentation and radical formation (Dy1^*^ + Dy2^*^). In the presence of molecular oxygen, reactive oxygen species can form, such as the superoxide anion (O_2_^*−^) [9]:

Dy + *hν* → ^#^Dy (1)

^#^Dy → Dy1^*^ + Dy2^*^ (2)

^#^Dy + O_2_ → Dy^*+^ + O_2_^*−^ (3)

By coupling UV with hydrogen peroxide (H_2_O_2_), the following chain mechanism is obtained [10,11]:

H_2_O_2_ + *hν* → 2 ^*^OH (4)

H_2_O_2_ + ^*^OH → HO_2_^*^ + H_2_O (5)

H_2_O_2_ + HO_2_^*^ → H_2_O + O_2_ + ^*^OH (6)

^*^OH + ^*^OH → H_2_O_2_ (7)

^*^OH + HO_2_^*^ → H_2_O + O_2_ (8)

HO_2_^*^ + HO_2_^*^ → H_2_O_2_ + O_2_ (9)

By coupling UV with persulfate anion (S_2_O_8_^2−^), the following chain mechanism is obtained [11,12,13]:

S_2_O_8_^2−^ + *hν* → 2 SO_4_^*−^ (10)

H_2_O + SO_4_^*−^ → HSO_4_^−^ + ^*^OH (11)

S_2_O_8_^2−^ + SO_4_^*−^ → S_2_O_8_^*−^ + SO_4_^2−^ (12)

^*^OH + S_2_O_8_^2−^ → S_2_O_8_^*−^ + OH^−^ (13)

^*^OH + ^*^OH → H_2_O_2_ (14)

SO_4_^*−^ + ^*^OH → HSO_5_^−^ (15)

SO_4_^*−^ + SO_4_^*−^ → S_2_O_8_^2−^ (16)

Coupling UV with H_2_O_2_ and S_2_O_8_^2−^ will generate the hydroxyl radical and, in the case of S_2_O_8_^2−^, the sulfate radical, and these radical species are active oxidants in the dyes’ degradation processes [11]. Besides these two homogeneous catalysts, the heterogeneous photocatalyst and semiconductor TiO_2_ is also commonly used as an AOP, and hydroxyl radical is produced according to the following mechanism [14,15,16,17,18]:

TiO_2_ + *hν* → TiO_2_ (e_CB−_ + h_VB+_) (17)

TiO_2_(h_VB+_) + H_2_O → TiO_2_ + H^+^ + ^*^OH (18)

TiO_2_(h_VB+_) + OH^−^ → TiO_2_ + ^*^OH (19)

TiO_2_(e_CB−_) + O_2_ → TiO_2_ + O_2_^*−^ (20)

After TiO_2_ UV absorption, an electron (e_CB−_) in the valence band is transferred to the conduction band, leaving a hole in the valence band (h_VB+_). The TiO_2_(h_VB+_) will oxidize water, and TiO_2_(e_CB−_) will reduce oxygen-generating reactive oxygen species such as hydroxide and superoxide radicals.

Nanomaterials are being coupled with UV-based AOP to make treatment processes more sustainable, i.e., to eliminate or reduce the amount of chemical substances involved in those processes, while increasing the pollutants degradation yields [6]. Taking into consideration natural resources, environmental sustainability, and toxicity issues, carbon-based nanomaterials are being preferred to metallic/semi-metallic materials in real-world applications [19]. Among the carbon-based nanomaterials, the synthetic versatility of carbon dots (CDs) [20,21,22,23,24,25,26,27,28,29] makes them the preferable choice to be coupling to an AOP for the treatment of textile wastewater.

Carbon dots are synthesized to achieve a desirable reactivity. In this paper, we describe the synthesis of a CD to be a catalyzer of the dye decomposition upon UV coupled with H_2_O_2_ and S_2_O_8_^2−^ AOPs. Because we were developing catalytic-based AOP, the objective was to increase the concentration of the hydroxyl radical and improve the dye decomposition or wastewater discoloration. In this work, the rational for the synthesis design had the following criteria:

1. To promote the decomposition of an oxidant molecule such as H_2_O_2_ and S_2_O_8_^2−^, an antioxidant is necessary. We have been using L-cysteine as a carbon precursor in CD synthesis [5]. Moreover, due to the existence of SH functional groups, it has antioxidant properties [30];
2. To increase the surface reactivity of the CD, the doping with a semiconductor such as TiO_2_, which is excited when exposed to UV light, was used. Indeed, this strategy has already been used for the production of photocatalytic carbon-based nanoparticles [31,32,33].
3. Polyethylene glycol (PEG) has been used in CD synthesis to increase the water solubility and to coat the CD with a soft surface [5]. Indeed, PEG undergoes caramelization, leading to the production of PEGylated nanoparticles [7,34,35,36,37].

In this paper, we describe the synthesis of two titanium(IV)-doped carbon-based nanomaterials (carbon dot), one with PEG (TiP-CD) and another without PEG (Ti-CD), that were used as catalysts in the UV based AOP discoloration of the anthraquinone dye, the Remazol Brilliant Blue R (also known as Reactive Blue 19) (RBB-R). Moreover, the performance of UV-based AOP (UV, UV/H_2_O_2_, UV/S_2_O_8_^2−^, and UV/TiO_2_) was assessed in the discoloration of aqueous solutions of azo dyes, methyl orange (MO), orange II sodium salt (O-II), and reactive black 5 (RB-5), besides RBB-R.

## 2. Materials and Methods

### 2.1. Reagents

**Figure 1:**
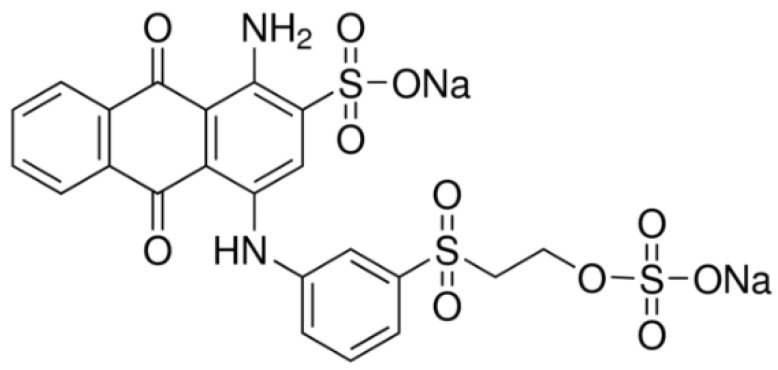
Chemical structure of Remazol Brilliant Blue R (Reactive Blue 19) (RBB-R).

Polyethylene glycol with an average molecular weight of 200 (PEG) (Sigma-Aldrich, St. Louis, MO, USA, P3015), L-cysteine (Sigma-Aldrich, St. Louis, MO, USA, W326305), titanium(IV) isopropoxide (Aldrich, St. Louis, MO, USA, 87560), ethylenediamine (Aldrich, St. Louis, MO, USA, E2,626-6), titanium dioxide (Sigma-Aldrich, St. Louis, MO, USA, anatase nanopowder <25 nm, 637254), hydrogen peroxide (Chem-Lab, Zedelgem, Belgium, CL00.2308), and sodium persulfate (Sigma-Aldrich, St. Louis, MO, USA, 216232) were used. The dyes used in this work were similar to those described in reference [5]. The chemical structure of the anthraquinone dye RBB-R is shown in Figure 1.

### 2.2. Synthesis of Titanium(IV)-Doped Carbon Dots (Ti-CD)

A one-pot button-up hydrothermal process was used for the Tip-CD and Ti-CD synthesis. The following solution was prepared for hydrothermal treatment: 0.400 g of L-cysteine was dissolved in 30 mL of water; after, only for the Tip-CD sample, 6.00 mL of PEG was used, and 0.60 mL of ethylenediamine was added and homogenized; lastly, 1.00 mL of titanium(IV) isopropoxide was added to the previous solution. The resulting mixture was placed in a Parr Series 4700 Pressure Vessel and treated hydrothermally for 5 h at 200 °C. The purification procedure was similar to that described in Reference [5].

### 2.3. UV Advanced Oxidation Process (AOP)

A 254 nm UV lamp system for water disinfection (25 W low-pressure mercury lamp S463RL, UV model S5Q-PA/2, VIQUA, Guelph, ON, Canada) was used. Experiments were carried out at pH = 3.00, 7.00, and 10.00, using the same methodology as described in Reference [5]. At the end of the experiments, the pH of the treated solutions was measured, and at pH = 3 experiments, it remained constant, but, at the pH = 7 and 10 experiments, it decreased to about 1 pH unit.

The initial concentration of the dye aqueous solutions was similar to that described in Reference [5]. For the analysis of the effect of the Tip-CD and Ti-CD, 0.500 mL of the solution was used.

### 2.4. Equipment

The synthesized carbon dots were characterized by using the following the equipment. Fluorescence analysis was measured in a 10 mm fluorescence quartz cell by using a Horiba Jobin Yvon FluoroMax spectrofluorimeter (New Jersey, NJ, USA), using 5 nm slit widths. The Zeta Potential was measured by using a particle analyzer Anton Paar Litesizer^TM^ 500 (Graz, Austria) and a polycarbonate Omega Cuvette (Ref. 155765). Fourier-transform infrared (FTIR) spectra were measured with a Perkin-Elmer (Waltham, MA, USA) Spectrum Two with an ATR sampling accessory. UV–Vis spectra were acquired with a VWR UV3100-PC spectrophotometer (Avantor, Radnor, PA, USA). AFM analysis was carried out by using a Veeco (Plainview, NY, USA) Metrology Multimode/Nanoscope IVA by tapping mode, using a Bruker (Billerica, MD, USA) silicon probe (model TESP-SS, resonant frequency of 320 kHz, nominal force constant of 42 N/m, and estimated tip radius of 2 nm). Scanning electron microscopy, SEM, was performed with a Quanta 200 from FEI Company (Hillsboro, OR, USA) coupled with energy dispersive spectroscopy (SEM–EDS).

### 2.5. Data Analysis

The registry of the absorbance of the dyes’ aqueous solutions as a function of the time of exposition to the UV light was carried out at the maximum absorbance of the dyes [5]. The percentage of dye removal (%DR), the apparent pseudo-first-order rate constant (*k*_ap_ without catalyst, *k*_ap_^c^ with H_2_O_2_, Na_2_S_2_O_8_, or TiO_2_ catalyst and *k*_ap_^N^ with nanomaterial), the percentage of increase of the rate constant when the catalyst is present (%Inc) and the percentage of increase of the rate constant when the nanomaterial is present (%Inc^N^) were calculated by using Equations (21)–(24), respectively (a linear relation between the absorbance and the aqueous concentration of the dyes was observed):

%DR = 100 × (Abs_0_−Abs_t_)/Abs_0_ (21)

ln(Abs_t_/Abs_0_)= − *k*_ap_
*t* (22)

%Inc = 100 × (*k*_ap_^c^ − *k*_ap_)/*k*_ap_ (23)

%Inc^N^ = 100 × (*k*_ap_^N^ − *k*_ap_^c^)/*k*_ap_^c^ (24)

where Abs_0_ and Abs_t_ are the absorbances of the dye aqueous solution at initial and *t* time of UV exposition.

## 3. Results

### 3.1. UV AOP of Dyes

**Figure 2:**
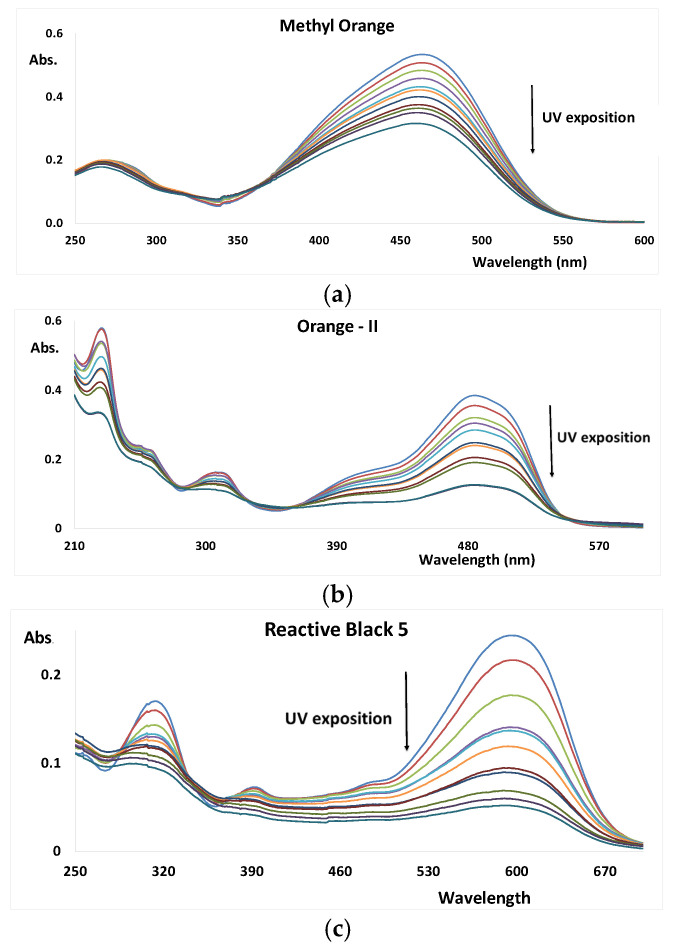

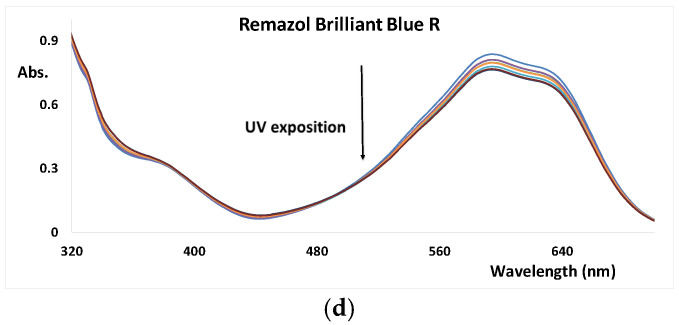
UV–Vis spectra of dyes as function of the UV exposition (pH = 10, and reaction time = 30 min). (**a**) Methyl orange; (**b**) Orange-II; (**c**) Reactive Black 5; and (**d**) Remazol Brilliant Blue R.

The UV exposition of the aqueous solutions of the dyes resulted differently for the four dyes and pH values under analysis (Figure 2 shows an example of the pH = 10 experiments). The analysis of the spectra as function of the time of UV exposition shows that the visible part of the spectra decreases the absorbance (discoloration), but, in the UV section of the spectra, absorption bands show different trends, suggesting the formation followed by degradation of other intermediate molecular fragments. Table 1 presents the discoloration kinetics parameters resulting from the UV AOP of the aqueous solutions of the dyes at three pH values: 3, 7, and 10.

The analysis of Table 1 shows that the discoloration apparent rate constants of the azo dyes are about ten times bigger than those of RBB-R. Moreover, for the two azo dyes, O-II and RB-5, the discoloration rate is bigger at pH = 10, with a rate constant value of about 0.04 min^−1^ and with a percentage of dye removal of about 70% in 30 min. The third azo dye show a lower decomposition time and has the biggest rate constant at pH = 3, at about 0.03 min^−1^, with a %DR of about 60% in 30 min. The anthraquinone dye RBB-R has a relatively lower rate constant, as can clearly be observed in Figure 2, achieving a maximum %DR of about 20% (30 min) at pH = 3.

In order to try to improve the decomposition rates of the dyes, UV was coupled with H_2_O_2_, S_2_O_8_^2^^−^, and TiO_2_. Because the pH of wastewaters of textile is close to neutral [5], the following work focused on aqueous solutions at pH = 7.

### 3.2. UV/H_2_O_2_ AOP of Dyes

**Figure 3:**
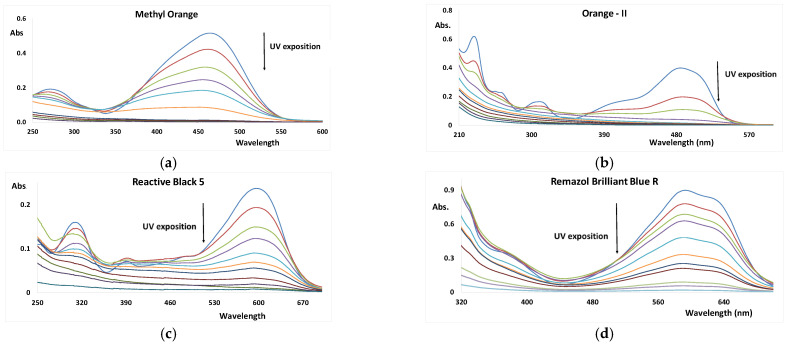
UV–Vis spectra of dyes as function of the UV/H_2_O_2_ exposition (pH = 7, and reaction time = 30 min). (**a**) Methyl orange; (**b**) Orange-II; (**c**) Reactive Black 5; and (**d**) Remazol Brilliant Blue R.

Table 2 and Figure 3 show the results obtained for the four dyes under the UV + H_2_O_2_ AOP. Coupling UV with a relatively low concentration of hydrogen peroxide of 0.3 mM allowed the AOP to reach almost complete discoloration of the azo dyes, but within different exposition times: MO, 30 min; O-II, 10 min; and RB-5, 4 min. However, at this H_2_O_2_ concentration, the decomposition of the RBB-R achieved only about 14% in 30 min. For the decomposition of RBB-R to reach about 96%, it was necessary to use a 100-times-higher H_2_O_2_ concentration.

The discoloration-rate values of the dyes studied in this work are higher than most results reported in the literature for the same dyes when UV/H_2_O_2_ AOP was used. One study evaluated the effectiveness of RB-5 bleaching through the UV/H_2_O_2_ system, in which a *k*_ap_ value of 0.105 min^−1^ was obtained (pH = 7) [38]. The degradation of MO was studied by using a UV/H_2_O_2_ process, and the almost complete degradation of MO was achieved in just 3 min when 0.1 mL of H_2_O_2_ was added to 20 mL of MO solution [39]. Four Fe-containing materials were used as heterogeneous photochemical catalysts for the discoloration of O-II in the presence of H_2_O_2_ and UV-C (pH values of 3 and 6), and the *k*_ap_ increased from 0.050 to 0.193 min^−1^ [40]. The discoloration of RB-5 was investigated through the UV/H_2_O_2_ process, which resulted in *k*_ap_ values ranging from 0.01 to 0.042 min^−1^ [41]. The discoloration capacity of RB-5 was evaluated through the advanced photochemical process ferrioxalate/H_2_O_2_/UV-C, in which a *k*_ap_ value of 0.0922 min^−1^ was obtained [42].

### 3.3. UV/S_2_O_8_^2−^ AOP of Dyes

Table 3 shows the results obtained for the four dyes under the UV + Persulfate AOP. Coupling UV with a concentration of persulfate higher than 0.1 mM allowed the AOP of the azo dyes to reach a discoloration percentage higher than 90% (at 30 min). However, for the decomposition of RBB-R to reach about 90%, an 80-times-higher persulfate concentration is necessary. This result shows that a similar relatively low efficiency, when compared with the azo dyes, for the RBB-R degradation is achieved with the UV/persulfate AOP when compared with the UV/H_2_O_2_ AOP.

The discoloration-rate values of the dyes, as shown in Table 3, are higher than most results reported in the literature for the same dyes when using AOPs based on the sulfate radical: namely, a UV/persulfate process was used for the degradation of MO, with a *k*_ap_ of 0.7 min^−1^ (25 °C) after 15 min [43]; the degradation of O-II by the AOP based on sulfate radical mediated by Fe^o^@Fe_x_O_y_ particles was investigated, and a *k*_ap_ of 0.057 min^−1^ was obtained [44]; Fe_3_O_4_ magnetic nanoparticles were used to activate peroxymonosulfate (PMS) in the degradation of RB-5, and when the PMS dosage increased from 0.25 to 2 mM, the kinetic rate constant showed the same trend, with an increase of 0.0085 min^−1^ to 0.0387 min^−1^ [45]; for the degradation of O-II, copper phosphide (Cu_3_P) was used as a heterogeneous catalyst for the activation of peroxydisulfate (PDS), and the *k*_ap_ was 0.1306 min^−1^ (pH = 7) [46]; and a persulfate system, activated by a nanocompound (α-FeOOH@GCA + K_2_S_2_O_8_), was applied for the discoloration of O-II, and a *k*_ap_ of 0.0502 min^−1^ was obtained [47].

### 3.4. UV/TiO_2_ AOP of Dyes

Table 4 shows the results obtained for the four dyes under the UV + TiO_2_ AOP, using a concentration of 0.050 g of TiO_2_/L. Comparing these results with the simple UV AOP, we see that large increases (between 151 and 331) in the discoloration are observed for the azo dyes. On the contrary, the presence of TiO_2_ almost does not affect the discoloration of RBB-R.

As shown in Table 4, the presence of TiO_2_ increased the rate of discoloration of the dyes. The catalytic effect of the UV/TiO_2_ AOP is well-known, and, for the dyes under investigation, the following results from the literature can be found: the effectiveness of RB-5 bleaching through the UV/TiO_2_ system had a *k*_ap_ value of 0.033 min^−1^ (pH = 7) [38]; TiO_2_ nanoparticles (NPs) were used in the degradation of MO under UV radiation, and the *k*_ap_ ranged from 0.005 to 0.0063 min^−1^ [48]; one study demonstrated efficient degradation of the O-II and RB-5 dyes through the UV/TiO_2_ photocatalytic system, with a *k*_ap_ of 0.025 and 0.005 min^−1^, respectively [49]; the photocatalytic discoloration of RB-5 in the presence of TiO_2_ nanoparticles under UV radiation was studied, and the *k*_ap_ values ranged from 0.01 to 0.125 min^−1^ [50]; a photocatalytic reactor with an ozone-generating mercury vapor lamp and TiO_2_ was used for the degradation of MO, and the *k*_ap_ was 0.0473 min^−1^ [51]; and the photocatalytic degradation of RBB-R was studied with thin films of nitrogen-doped TiO_2_ catalysts under UV-A radiation, and the values were *k*_ap_ = 5.1 ×10^−5^ (undoped), *k*_ap_ = 1.8 × 10^−4^ (doped with 0.72% of N), and *k*_ap_ = 2.0 × 10^−4^ (doped with 0.84% of N) [52].

The above UV-based AOPs are not very effective for the anthraquinone dye RBB-R decomposition unless relatively high concentrations of hydrogen peroxide and persulfate are used; however, this raises sustainability problems. As an alternative, a carbon-based nanocatalyst was developed to be coupled to UV and hydrogen peroxide or persulfate to increase the efficiency of the RBB-R discoloration.

### 3.5. Preliminary Analysis of the Catalytic Performance of the Synthesized CD

The basic precursors of the CD developed in this project were L-cysteine and ethylenediamine, which were used as carbon and nitrogen sources, respectively. The other two components, PEG and the titanium compound, were analyzed for their essential role in the final photocatalyst. Taking into consideration the critical role of PEG previously observed in the ozonation catalyst [5], two CDs were synthesized with (TiP-CD) and without PEG (Ti-CD). The preliminary comparative analysis of the two photocatalysts confirmed that PEG is essential to the observed photocatalysis, and this observation is further supported below. In the case of titanium doping (namely TiO_2_), a CD was synthesized by using the composition described for TiP-CD (Section 2.2), but without the titanium compound. The catalytic discoloration properties of this non-doped CD was markedly worse than the TiP-CD. For this reason, the work focused mainly on TiP-CD investigation.

### 3.6. Characterization of the Nanomaterials

**Figure 4:**
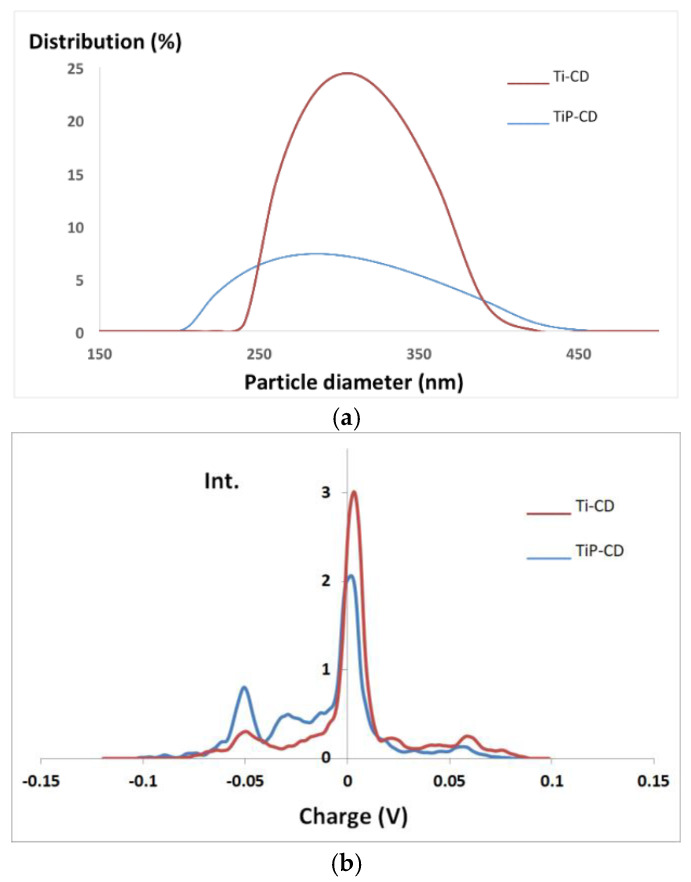
(**a**) DLS spectra and (**b**) Zeta potential of Ti-CD and TiP-CD.

**Figure 5:**
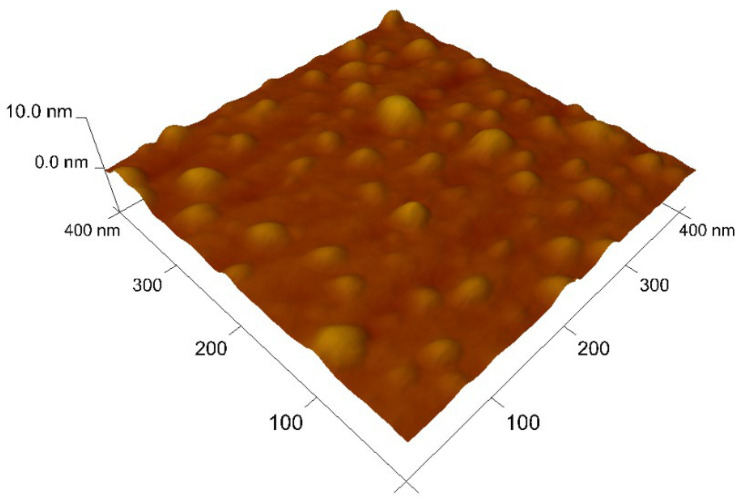
AFM image of TiP-CD.

In aqueous solution, the nanomaterials become agglomerated and hydrated, as seen the DLS results shown in Figure 4a, with an average hydrodynamic size of about 310 and 297 nm for Ti-CD and TiP-CD, respectively. Figure 4b shows that the mean Zeta potential is −0.3 and −3.5 mV for Ti-CD and TiP-CD, respectively, with a charge distribution ranging from slightly positively charged to slightly negatively charged nanoparticles. Figure 5 shows the AFM images of the TiP-CD. The analysis of the AFM results shows that they have a spherical shape, with an average size of 22 ± 3 nm.

**Figure 6:**
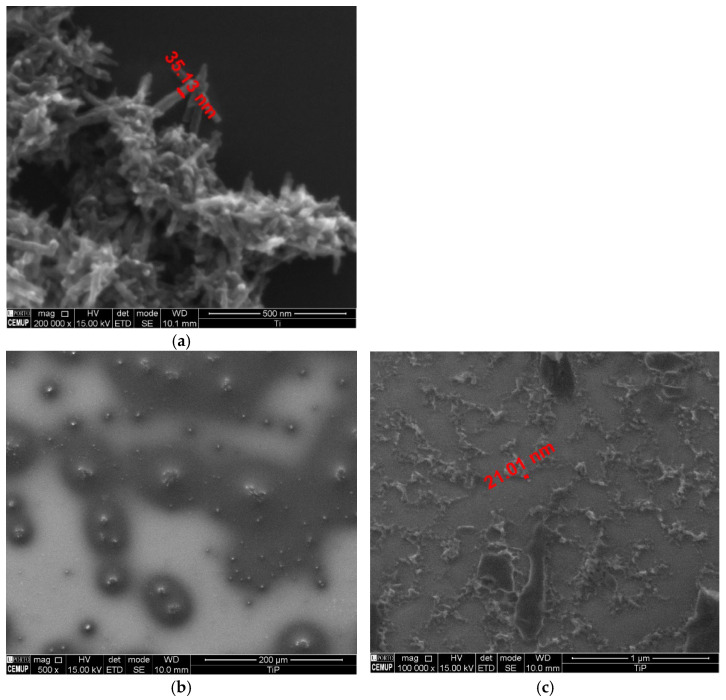

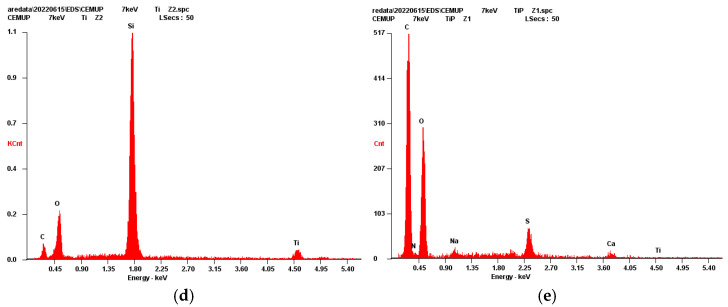
SEM images of the Ti-CD (**a**) and TiP-CD (**b**,**c**) samples and the corresponding EDS (Ti-CD (**d**) and TiP-CD (**e**)).

Figure 6 shows the SEM and EDS of Ti-CD (Figure 6a,d) and TiP-CD (Figure 6b,c,e) samples. The morphology of the two samples, as revealed by SEM, is rather different. While Ti-CDs (Figure 6a) have a nanotube shape, with a diameter of about 35 nm, and are highly agglomerated into bigger clusters, the TiP-CDs (Figure 6b,c) are dispersed with a PEG capping (particularly clear in Figure 6b) that confirm the size determined by AFM; Figure 6c shows as an example a nanoparticle with a size of 21 nm. This result shows the role of PEG on the capping, dispersion, and shape of the nanoparticles. The EDS of the two samples confirm the titanium doping, particularly in the Ti-CD sample (Figure 6d). In the case of TiP-CDs, because they are highly dispersed and capped with PEG, titanium is hardly detected, but, as expected, the presence of carbon, oxygen, and sulfur is clearly observed.

**Figure 7:**
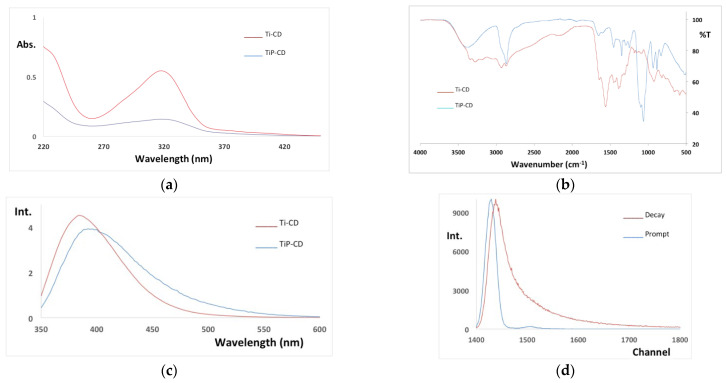
(**a**) UV–Vis, (**b**) FTIR, (**c**) fluorescence emission (ex. 350 nm) spectra of Ti-CD and TiP-CD, and (**d**) fluorescence lifetime decay.

Figure 7 shows the UV–Vis (Figure 7a), FTIR (Figure 7b), and fluorescence emission at the maximum of the excitation (325 nm) (Figure 7c) spectra of both the CD and the lifetime decay of TiP-CD (Figure 7d). The UV–Vis spectra of both samples (Figure 5a) are characterized by a main band at 317 nm and a shoulder at 286 nm. These spectra are similar to other CDs’ spectra, with a strong absorption in the ultraviolet that tails into the visible region due to the n–χ* transition of multi-conjugate C=O [53,54].

The FTIR spectra of Ti-CD and TiP-CD (Figure 7b) are rather different due to the PEG capping of sample TiP-CD. The spectrum of Ti-CD is characterized by the following main bands [53,54,55]: C=O stretching can be observed at 1652 cm^−1^; C–C stretching bands are located at 1568 and 1461 cm^−1^; the bands at 3205, 3300, and 3362 cm^−1^ are related to the stretching vibration of N–H and O–H bonds; the bands at 1397 and 1386 cm^−1^ are attributed to the bending modes of O–H and/or C–H; and the stretching vibration of C–H bonds appeared at 2947 and 2884 cm^−1^. The FTIR spectrum of TiP-CD is dominated by the bands due to PEG [56,57]: C–O and C–C stretching and CH_2_ rocking at 841 cm^−1^; CH_2_ rocking and CH_2_ twisting at 939 cm^−1^; C–O and C-C stretching and CH_2_ rocking at 1066 cm^−1^; C–O and C–C stretching at 1105 cm^−1^; C–O stretching and CH_2_ rocking at 1105 cm^−1^; CH_2_ twisting at 1226 cm^−1^; CH_2_ wagging at 1353 cm^−1^; CH_2_ scissoring at 1461 cm^−1^; C-H stretching at 2896 cm^−1^; and O-H stretching at 3418 cm^−1^.

The fluorescence emission spectra characterized by a broad band with a maximum at 384 and 394 nm for Ti-CD and TiP-CD, respectively, that are responsible for a typical blue emission of CD [58,59,60]. The lifetime decay of the TiP-CD fits (with a χ^2^ = 1.25) a three-fluorophores model with the following lifetimes (pre-exponential factors under parenthesis): 0.69 ± 0.02 ns (0.0508 ± 0.0003), 2.67 ± 0.08 ns (0.0241 ± 0.0001), and 2.67 ± 0.08 ns (0.00583 ± 0.00003).

### 3.7. UV-Based AOP of RBB-R in the Presence of TiP-CD

**Figure 8:**
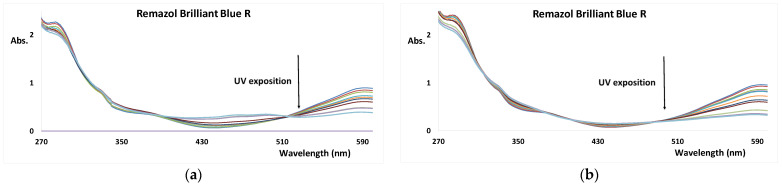
UV–Vis spectra of RBB-R as function of the (**a**) UV/H_2_O_2_/TiP-CD and (**b**) UV/S_2_O_8_^2^^−^/TiP-CD exposition (pH = 7, and reaction time = 30 min).

The effect of the addition of the TiP-CD in the UV/H_2_O_2_ and UV/persulfate degradation kinetics of the RBB-R dye is shown in Figure 8 and Table 5. The presence of Ti-CD or TiP-CD on the single UV AOP of RBB-R inhibits the discoloration rate. However, in the UV/H_2_O_2_ and UV/persulfate AOP, a marked increase in the discoloration rate is observed when TiP-CD is present, reaching a 263% increase. However, as expected from the initial catalytic evaluation of both nanomaterials, the effect of Ti-CD is null. This result supports the important role of the PEG capping in the nanocatalysts of AOP [5].

Taking into consideration that the decomposition of the dyes occurs due to the reaction with the hydroxyl (mainly Equations (4), (6), (11), and (18)) and sulfate (Equation (10)) radicals, the presence of TiP-CD will somehow promote the formation of these highly oxidant species. The enhancement of photodegradation of RBB-R in the presence of TiP-CD and H_2_O_2_ or S_2_O_8_^2−^ may be attributed to the combined effects of photodissociation of the oxidant species in the presence of UV light and TiP-CD surface, the presence of the hydroxyl radical species on the reactant mixture, and the PEG coating together with the dye [61]. A hypothesis of the direct role of TiP-CD may be in the surface catalyzed hydroxyl radical formation, as described in Equation (25) for the case of H_2_O_2_ [62]:

TiP-CD + H_2_O_2_ → TiP-CD^+^ + OH^−^ + *OH (25)

## 4. Conclusions

A titanium(IV)-doped and PEG-capped carbon dot synthesized from L-cysteine and ethylenediamine (TiP-CD) showed itself to be an efficient catalyst for the UV/H_2_O_2_ and UV/persulfate AOP. Indeed, marked increases of up to 263% of the discoloration rate of the anthraquinone dye RBB-R were observed when the TiP-CD was present; this can open up new sustainable AOP strategies for the degradation of families of dyes that are difficult to degrade. Discoloration pseudo-first-order rate constants in the order of 0.034 min^−1^ were obtained with both UV/H_2_O_2_ and UV/persulfate AOP systems.

AOP, UV/H_2_O_2_, and UV/persulfate generate the hydroxyl radical upon UV exposition, and, in the case of persulfate, the radical sulfate, the observed catalytic role of the carbon-based nanomaterial is related to an increase of the hydroxyl radical production. Moreover, because PEG is crucial for the observed catalytic effect, and PEG is capping the nanoparticle, the active substrate, H_2_O_2_, and persulfate anion will become trapped in the gelified external layer of the nanoparticle, and the UV-activated core will decompose the substrate into hydroxyl and sulfate radicals.

## Figures and Tables

**Table 1:** Apparent rate constant and percentage of dye removal (at 30 min) of the UV AOP of dyes (the standard deviations of at least three independent repetitions are shown). The fitting of the integrated first-order kinetics resulted in R^2^ > 0.98.

Dye	pH	*k*_ap_ (min^−1^)	%DR
**MO**	3	0.0294 ± 0.0108	63.53 ± 3.56
	7	0.0051 ± 0.0006	15.23 ± 1.94
	10	0.0169 ± 0.0012	40.58 ± 0.27
**O-II**	3	0.0129 ± 0.0003	33.73 ± 0.83
	7	0.0133 ± 0.0016	32.53 ± 3.16
	10	0.0337 ± 0.0006	64.50 ± 2.57
**RB-5**	3	0.0227 ± 0.0006	47.37 ± 3.42
	7	0.0235 ± 0.0007	48.39 ± 1.94
	10	0.0474 ± 0.0046	78.07 ± 1.03
**RBB-R**	3	0.0078 ± 0.0009	22.20 ± 3.10
	7	0.0034 ± 0.0003	9.16 ± 0.32
	10	0.0025 ± 0.0009	7.78 ± 2.82

**Table 2:** Apparent first-order rate constant, percentage of the rate constant when the catalyst is present, and percentage of dye discoloration of the UV + H_2_O_2_ AOP of dyes (the standard deviations of at least three independent repetitions are shown). In parentheses, the reaction time necessary to achieve the percentage of dye removal is shown. The fitting of the integrated first-order kinetics resulted in R^2^ > 0.98.

Dye	*k*_ap_^c^ (min^−1^)	%Inc	%DR
MO + 0.30 mM H_2_O_2_	0.2294 ± 0.0179	4398	99.95 ± 0.03 (30 min)
O-II + 0.30 mM H_2_O_2_	0.5921 ± 0.1465	4352	98.64 ± 0.46 (10 min)
RB-5 + 0.30 mM H_2_O_2_	0.9017 ± 0.0773	3737	96.19 ± 1.93 (4 min)
RBB-R + 0.30 mM H_2_O_2_	0.0046 ± 0.0007	35	13.87 ± 1.84 (30 min)
RBB-R + 3.0 mM H_2_O_2_	0.0091 ± 0.0016	168	23.72 ± 1.76 (30 min)
RBB-R + 30 mM H_2_O_2_	0.1169 ± 0.0121	3338	96.22 ± 1.43 (30 min)

**Table 3:** Apparent first-order rate constant, percentage of the rate constant when the catalyst is present, and percentage of dye discoloration of the UV + S_2_O_8_^2−^ AOP of dyes (the standard deviations of at least three independent repetitions are shown). In parentheses, the reaction time necessary to achieve the percentage of dye removal is shown. The fitting of the integrated first-order kinetics resulted in R^2^ > 0.98.

Dye	*k*_ap_^c^ (min^−1^)	%Inc	%DR
MO + 0.10 mM Na_2_S_2_O_8_	0.0677 ± 0.0010	1227	87.05 ± 0.78 (30 min)
MO + 1.02 mM Na_2_S_2_O_8_	0.9365 ± 0.0810	18,263	98.56 ± 0.60 (5 min)
O-II + 0.010 mM Na_2_S_2_O_8_	0.0255 ± 0.0205	400	54.08 ± 0.42 (30 min)
O-II + 0.10 mM Na_2_S_2_O_8_	0.4498 ± 0.0280	8720	94.99 ± 3.39 (30 min)
O-II + 1.02 mM Na_2_S_2_O_8_	1.1187± 0.1949	21,835	98.86 ± 0.31 (5 min)
RB-5 + 0.010 mM Na_2_S_2_O_8_	0.0601 ± 0.0042	1078	80.74 ± 4.24 (30 min)
RB-5 + 0.10 mM Na_2_S_2_O_8_	0.5608 ± 0.0606	10,896	91.03 ± 4.68 (5 min)
RBB-R + 0.010 mM Na_2_S_2_O_8_	0.0017 ± 0.0049	−50	5.31 ± 1.21 (30 min)
RBB-R + 1.02 mM Na_2_S_2_O_8_	0.0277 ± 0.0049	715	58.70 ± 7.36 (30 min)
RBB-R + 2.04 mM Na_2_S_2_O_8_	0.0531 ± 0.0108	1462	80.39 ± 5.93 (30 min)
RBB-R + 4.07 mM Na_2_S_2_O_8_	0.0712 ± 0.0024	1994	81.59 ± 4.87 (30 min)
RBB-R + 8.15 mM Na_2_S_2_O_8_	0.0960 ± 0.0056	2724	95.25 ± 1.21 (30 min)

**Table 4:** Apparent rate constant, percentage of the rate constant when the catalyst is present, and percentage of dye removal of the UV + TiO_2_ AOP of dyes (the standard deviations of at least three independent repetitions are shown). In parentheses, the reaction time necessary to achieve the percentage of dye removal is shown. The fitting of the integrated first-order kinetics resulted in R^2^ > 0.98.

Dye	*k*_ap_^c^ (min^−1^)	%Inc	%DR
MO + 0.050 g/L TiO_2_	0.0220 ± 0.0032	331	48.92 ± 5.28 (30 min)
O-II + 0.050 g/L TiO_2_	0.0409 ± 0.0022	208	68.69 ± 1.96 (30 min)
RB-5 + 0.050 g/L TiO_2_	0.0591 ± 0.0085	151	66.12 ± 2.22 (30 min)
RBB-R + 0.050 g/L TiO_2_	0.0035 ± 0.0009	3	8.87 ± 1.29 (30 min)

**Table 5:** Apparent rate constant, percentage of the rate constant when Ti-CD is present, and percentage of dye removal (at 30 min) of the UV + Ti-CD AOP of RBB-R (the standard deviations of at least three independent repetitions are shown). The fitting of the integrated first-order kinetics resulted in R^2^ > 0.98.

	*k*_ap_^N^ (min^−1^)	%Inc^N^	%DR
RBB-R + TiP-CD	0.0029 ± 0.0003	−14	8.02 ± 0.88
RBB-R + Ti-CD	0.0023 ± 0.0003	−32	7.17 ± 0.71
RBB-R + 0.30 mM H_2_O_2_ + TiP-CD	0.0071 ± 0.0021	54	17.83 ± 5.29
RBB-R + 3.0 mM H_2_O_2_ + TiP-CD	0.0330 ± 0.0087	263	57.91 ± 8.91
RBB-R + 3.0 mM H_2_O_2_ + Ti-CD	0.0079 ± 0.0013	1.3	21.12 ± 3.51
RBB-R + 0.010 mM Na_2_S_2_O_8_ + TiP-CD	0.0021 ± 0.0001	24	6.18 ± 0.40
RBB-R + 1.02 mM Na_2_S_2_O_8_ + TiP-CD	0.0345 ± 0.0032	25	63.35 ± 1.99
RBB-R + 1.02 mM Na_2_S_2_O_8_ + Ti-CD	0.0236 ± 0.0014	6	52.59 ± 0.65

## Data Availability

Not applicable.
